# Antifungal and Antitoxigenic Effects of Selected Essential Oils in Vapors on Green Coffee Beans with Impact on Consumer Acceptability

**DOI:** 10.3390/foods10122993

**Published:** 2021-12-04

**Authors:** Miroslava Hlebová, Lukas Hleba, Juraj Medo, Viktoria Uzsakova, Pavel Kloucek, Matej Bozik, Peter Haščík, Juraj Čuboň

**Affiliations:** 1 Department of Biology, Faculty of Natural Sciences, University of SS. Cyril and Methodius, Nám. J. Herdu 2, 917 01 Trnava, Slovakia; 2 Institute of Biotechnology, Faculty of Biotechnology and Food Sciences, Slovak University of Agriculture, Nitra, Tr. A. Hlinku 2, 949 76 Nitra, Slovakia; lukas.hleba@uniag.sk (L.H.); juraj.medo@uniag.sk (J.M.); viktoria.uzsakova@uniag.sk (V.U.); 3 Department of Food Science, Faculty of Agrobiology, Food and Natural Resources, Czech University of Life Sciences Prague, Kamycka 129, 165 00 Prague–Suchdol, Czech Republic; kloucek@af.czu.cz (P.K.); bozik@af.czu.cz (M.B.); 4 Institute of Food science, Faculty of Biotechnology and Food Sciences, Slovak University of Agriculture, Nitra, Tr. A. Hlinku 2, 949 76 Nitra, Slovakia; peter.hascik@uniag.sk

**Keywords:** antifungal activity, essential oils, *Aspergillus*, coffee, ochratoxin A, aflatoxins

## Abstract

The main objective of this study is to evaluate the effect of selected essential oils thyme chemotype linalool (*Thymus zygis* L.), thyme chemotype tymol (*Thymus vulgaris* L.), eucalyptus *(Eucalyptus globulus* Labill.), lavender (*Lavandula angustifolia* Mill.), mint (*Mentha piperita* L.), almond (*Prunbus dulcis* Mill.), cinnamon bark (*Cinnamomum zeylanicum* Nees), litsea (*Litsea cubeba* Lour. Pers), lemongrass (*Cympogon citrati* L. Stapf), and ginger *(Zingiber officinalis* Rosc.) in the vapor phase on growth, sporulation, and mycotoxins production of two *Aspergillus* strains (*Aspergillus parasiticus* CGC34 and *Aspergillus ochraceus* CGC87), important postharvest pathogens of green and roasted coffee beans. Moreover, the effect of the essential oils (EOs) on the sensory profile of the coffee samples treated with EOs was evaluated. The major components of tested EOs were determined by gas chromatography and mass spectrometry (GC–MS) and gas chromatography with flame ionization detector (GC-FID). The results showed that almond, cinnamon bark, lemongrass, and litsea EOs are able to significantly inhibit the growth, sporulation, and mycotoxins production by toxigenic fungi. Sensory evaluation of coffee beans treated with EOs before and after roasting showed that some EOs (except lemongrass and litsea) do not adversely affect the taste and aroma of coffee beverages. Thus, application of the vapors of almond and cinnamon EOs appears to be an effective way that could serve to protect coffee during its transport and storage from toxigenic fungi.

## 1. Introduction

Coffee is one of the most consumed and wanted beverages all over the world, due to its specific aroma and flavor and also for the beneficial effects of caffeine, which improves alertness and stimulates physical performance [1,2]. Despite the unique properties of this beverage, coffee could represent a health threat, especially to the frequent consumer of coffee [3]. The safety of coffee and coffee products is affected by microbial contamination because coffee cherries and beans such as other crops are exposed and highly susceptible to contamination during different phases of their processing (plant development, harvesting, transport, storage) [4]. Among spoilage agents, filamentous fungi pose the highest risk to human health through their possibility to produce harmful secondary metabolites—mycotoxins [5]. The main toxigenic fungi contaminating coffee belongs to the genus *Aspergillus*; mycotoxins produced by this genus can cause minor to severe complications [6].

Ochratoxin A (OA) is major and the most studied mycotoxin in coffee worldwide [7]. Many microbial studies have also confirmed the presence of other important mycotoxins in coffee—aflatoxins (AFs). These mycotoxins display a vast toxicity (carcinogenic, nephrotoxic, immunotoxic, hepatotoxic, embryotoxic, teratogenic, and mutagenic) [8,9]. Based on their carcinogenic effects, AFs were classified by the International Agency for Research on Cancer (IARC) as human carcinogens (group 1) and OA as a possible carcinogen (group 2B) [10,11]. Despite their high toxicity, OA is the only one mycotoxin monitored at the coffee production, with maximal levels being 5 μg/kg (roasted coffee beans and ground roasted coffee) and 10 μg/kg (soluble coffee) [12].

There are currently certain pre- and postharvest strategies to protect coffee from contamination caused by microscopic fungi and their mycotoxins, especially OA. Preharvest contamination of coffee beans depends on their maturity (fragility, damage = infection) [13]. However, the results showed that more frequent infection of coffee beans with fungi occurs mainly after harvest (sources of fungal contamination: soil, the surface of the dryer equipment, etc.); so, adequate preharvest management only involves the use of quality and healthy coffee fruits [14]. In postharvest strategies, adequate harvesting techniques are fundamental as well as the drying process or the methods of wet and dry coffee beans processing. In addition, green coffee beans storage after processing is the most crucial phase of a strategy to protect the coffee from contamination [15]. During transport by ship (the most common transport of coffee), coffee should not have more than 12 to 14% humidity, and during transport in general, the relative humidity (should be between 50 and 75%) and the temperature (not exceeding 26 °C, the maximum limits for fungal growth) should be controlled [16]. However, condensation may occur during transport by ship, which promotes an increase in water activity (a_w_) in coffee by reabsorption, leading to fungal growth [17].

Therefore, enhancement of the quality and streamlining of coffee processing technology, as well as its storage and transport, is vital to prevent detrimental effects of contamination by microscopic fungi and their secondary metabolites. One possibility is using plant-based products—essential oils (EOs).

Essential oils and their bioactive components have shown a wide range of biological activity. EOs have antifungal, antiviral, antibacterial, antimutagenic, or antioxidant properties, and therefore, have a wide application in food industry and agriculture [18]. As essential oils are natural substances and can affect the growth, sporulation, and even the production of mycotoxins by filamentous fungi [19], they are promising and suitable alternatives to solve the problem of coffee contamination by fungi and their mycotoxins, as well as in other food commodities.

The effects of essential oils on contaminants directly on/in coffee beans are still limited. Therefore, in this study, we evaluate the effect of selected essential oils on growth, sporulation, and mycotoxins production of two *Aspergillus* strains, important postharvest pathogens of green and roasted coffee beans, as well as the effect of essential oil treatments on the sensory profile of coffee.

## 2. Materials and Methods

### 2.1. Fungal Strains Origin

Two isolates of the genus *Aspergillus*, namely, *A. ochraceus* (CGC87) and *A. parasiticus* (CGC34), were used. Both isolates were originally harvested from green coffee beans (*Coffea arabica* L.) originating in Colombia (obtained from the different coffee shops in Slovakia). The used isolates were selected based on their ability to produce mycotoxins (Table 1). All used fungal isolates were identified by ITS rDNA sequencing according to Císarová et al. [20]. The accession numbers of used strains (deposited in GenBank) are summarized in Table 1.

### 2.2. Essential Oils Used in Study

All ten essential oils, namely, thyme chemotype linalool (*Thymus zygis* L.), thyme chemotype tymol (*Thymus vulgaris* L.), eucalyptus *(Eucalyptus globulus* Labill.), lavender (*Lavandula angustifolia* Mill.), mint (*Mentha piperita* L.), almond (*Prunbus dulcis* Mill.), cinnamon bark (*Cinnamomum zeylanicum* Nees), litsea (*Litsea cubeba* Lour. Pers), lemongrass (*Cympogon citrati* L. Stapf), and ginger *(Zingiber officinalis* Rosc.) were obtained from commercial supplier Hanus—Bylinné prípravky (Hanus, Nitra). All tested EOs were obtained by hydrodistillation and stored in hermetically sealed glass vial at 4 °C in the dark, until use.

### 2.3. Chemical Analysis of Essential Oils

The chemical analysis of EOs were carried out by gas chromatography and mass spectrometry (GC–MS) using an Agilent 7890A GC (HPST, s.r.o., Praha, Czech Republic) coupled to an Agilent MSD5975C MS detector (Agilent Technologies, Palo Alto, CA, USA) with a HP-5MS column (30 m × 0.25 mm, 0.25 μm-film-thickness) (HPST, s.r.o., Praha, Czech Republic). Relative proportions of essential oils components were assessed by gas chromatography using a gas chromatograph with flame ionization detector (GC–FID) using Agilent 7890A (Agilent Technologies, Palo Alto, CA, USA) with HP-5MS, 30 m × 0.25 mm, 0.25 μm-film-thickness. Both analyses were prepared in the same way according to Božik et al. [21] and Klouček et al. [22]. The authentic standards (Sigma–Aldrich, Munich, Germany) were used for components identification. The identification was based on a comparison of the obtained mass spectra of the different components. The chemical components were quantified by dividing peak area by the total area of all peaks and major compounds (only peaks over 0.1% were counted).

### 2.4. Antifungal Analysis in Green Coffee

#### 2.4.1. Preparation of Fungal Inoculum

The used strains were cultivated on Sabouraud dextrose agar (SDA) (HiMedia, Mumbai. India) at 25 ± 1 °C in the dark until the analysis. Fungal inoculum was prepared in 20 mL of sterile distilled water from 5–7 days-old culture gently rubbed on the lawn surface with a spreader and harvested through sterile cheesecloth. The PBS (phosphate buffer saline) was applied to the culture surface along with 0.5% Tween 80 and spores were collected using a micropipette to a prepared and final concentration of 10^6^ CFU/mL. The inoculum density was adjusted to 0.7 to 1.2 McFarland units depending on the tested strain.

#### 2.4.2. Green Coffee Beans Inoculation and Treatment with Essential Oils

The same coffee bean varieties (*Coffea arabica* L.) from which the tested strains were originally isolated were used in this study. The inoculated coffee beans were prepared and treated with essential oils according to Božik et al. [21] with minor modification. Firstly, the green coffee beans were sterilized by 70% ethanol (30 s), then mixed in 3% solution of chloramine for 2 min and washed by sterile distilled water. A total 40 g of dry sterile coffee beans were transferred to an Erlenmeyer flask together with 20 mL of fungal inoculum suspension and then mixed for 20 min. The essential oils were diluted in ethyl acetate to final concentrations of 250, 125, 62.5, 31.5, and 15.625 μL/L of air. The sterile filter paper Whatman (Ø 60 mm) was placed on the Petri dish lid (Ø 90 mm) and 100 μL of essential oil suspension was evenly distributed on it. Plastic nets (Ø 90 mm) were placed in Petri dishes to prevent the coffee beans from coming into contact with the essential oil. Then, 7 g of dry inoculated coffee beans were placed into Petri dishes on a plastic net. The petri dish was sealed and allowed to cultivate at 50 °C for 2 h in an oven and then for 20 min at room temperature (22 °C). The filter paper with ethyl acetate alone served as a positive growth control. In order to exclude the negative effect of temperature on the growth and sporulation of microscopic fungi, only the inoculated coffee beans without the essential oils were used as a control. All analyses were performed in three replicates (3 × 12 pieces of coffee beans for each tested concentration; a total of 252 replicates for each tested essential oil + two control sets (ethyl acetate and temperature control) were used).

For evaluation of coffee beans treated with essential oils, 96-well microtiter plates were used. A total 100 μL SB medium was added to each well. Then, the coffee beans were placed into a 96-well microtiter plate (one coffee bean per well) together with the control samples (temperature control and ethyl acetate control) and cultivated for 7 days at 25 ± 1 °C in the dark. The last 12 wells of the 96-well microtiter plate contained only 100 μL of sterile SB and served as a medium purity control. After seven days, the wells with the visible mycelial growth and sporulation were counted.

#### 2.4.3. The Evaluation of Essential Oils Anti-Toxicogenic Effect

The effect of essential oils on the ability of microscopic fungi to produce mycotoxins, namely, aflatoxins B_1_ (AFB_1_), G_1_ (AFG_1_), and ochratoxin A (OA), were evaluated. All treated samples of coffee beans with control sets (with visible growth and sporulation) were removed from the 96-microtiter plate with sterile tweezers and placed on Petri dishes (Ø 60 mm) with SDA medium (one bean per Petri dish). The sealed Petri dishes were cultivated for the next 7 days at 25 ± 1 °C in the dark. After cultivation, the individual coffee beans were placed into a microtube to detect mycotoxins by thin layer chromatography, previously described in Labuda and Tančinová [23]. The resulting spots on the chromatographic plate were visualized and compared to authentic standards (Sigma Aldrich, Munich, Germany). Aflatoxins and ochratoxin spots were visualized under UV light at 365 nm (blue spot (R_f_ 0.65) for AFB_1_, green spot (R_f_ 0.39) for AFG_1_, and blue spots (R_f_ 0.45) for OA) [24].

#### 2.4.4. Preparation of Coffee Samples until Sensory Analysis

Prior to sensory analysis, the green coffee samples (the same varieties (*Coffea arabica* L.) as green coffee beans were used) were prepared in two ways due to the comparison of the EOs effects on the sensory quality of coffee before and after roasting.

First, noninoculated already roasted coffee beans (7 g) were treated with three concentrations (62.5, 125, and 250 μL/L of air) of the most effective EOs (as described in Section 2.4.2). After treatment in the oven (50 °C) and cooling to room temperature (22 °C), the coffee beans were stored in hermetically watertight bags for 7 days (without presence of EOs) until tasting. After the storage period, they were used for sensory evaluation.

Second, the noninoculated green coffee beans were treated with the vapor phase of the most effective essential oils according to Císarová et al. [25]. The green coffee samples (25 g) were hermetically sealed in 0.5 L sterile glass jars (Bromioli Rocco, Italy), which contained the sterile filter paper disc (60 mm) on the cover of the jar. Then, 100 μL of each EO diluted in ethyl acetate (62.5, 125, and 250 μL/L of air) was applied by the pipette on a sterile paper–filter disc. The glass jars were hermetically closed and then stored in the dark for 7 days. A minimum 250 g of coffee beans is required for the roasting process; therefore, a total of 10 glass jars were prepared for each concentration of each of the tested EOs. After 7 days of storage, the samples were removed from the glass jar and roasted for 24 h before sensory analysis. The humidity of the green coffee beans before the application of essential oils was 10.4% (humidity measured by the Lighttells MD 500) and the humidity of the green coffee beans before roasting ranged from 11.6% to 11.8%. The samples were roasted in a Hottop kn-8828b-2k+ roaster. The coffee beans were put to the roaster at an initial temperature of 104 °C; then, the temperature dropped to 57 °C (1.5 s), and next was increased to a maximum of 207 °C. The roasting time was 13 min and 38 s to achieve a medium dark color. After roasting, the coffee samples were cooled to room temperature (22 °C) and used for sensory evaluation.

### 2.5. Sensory Analysis of Roasted Coffee Treated by Essential Oils

Due to the possible negative influence of essential oils on the intensity of aroma and the acceptability of the taste of coffee beans, a sensory analysis of treated coffee beans with essential oils was performed. Noninoculated roasted coffee beans (treated with EOs (62.5, 125, and 250 μL/L of air)) in both ways as described in Section 2.4) were used for sensory evaluation. The essential oils and their concentrations were selected according to their ability to effectively inhibit the growth, sporulation, and mycotoxin production of the tested strains. Before preparing the coffee drinks, the samples were weighed in grains (7 g) and ground for 10 min. Then, they were put to glass cups and prepared for tasting by pouring 100 mL of hot water (97 °C) into it and, after 2 min, subsequently served to the evaluator. The untreated samples served as a control.

All sensory evaluations were made in a sensory evaluation laboratory according to ISO guidelines [26]. Sensory evaluation of treated coffee samples was determined by an inverted hedonic test based on a five-point scale only according to the odor and taste parameters of the samples. Samples were administered in order: first, the control sample; then, other samples treated with EOs (marketed with numbers) were provided to untrained evaluators (7 untrained evaluators aged 25 to 50). The task for the evaluators was to assign a value to the submitted sample based on their taste and olfactory preferences and to enter these values in the submitted form. Sensory properties evaluated include score for taste (5—excellent, intense; 4—pleasant, less intense; 3—less pleasant, less pronounced; 2—unpleasant, atypical; 1—very unpleasant) and for odor evaluation (5—very pleasant, 4—quite pleasant, 3—satisfactory, 2—still acceptable, 1—unpleasant). The maximum number of points that each sample of treated coffee could obtain was 35 points and a minimum was 7 points.

### 2.6. Statistical Analysis

**Figure 1:**
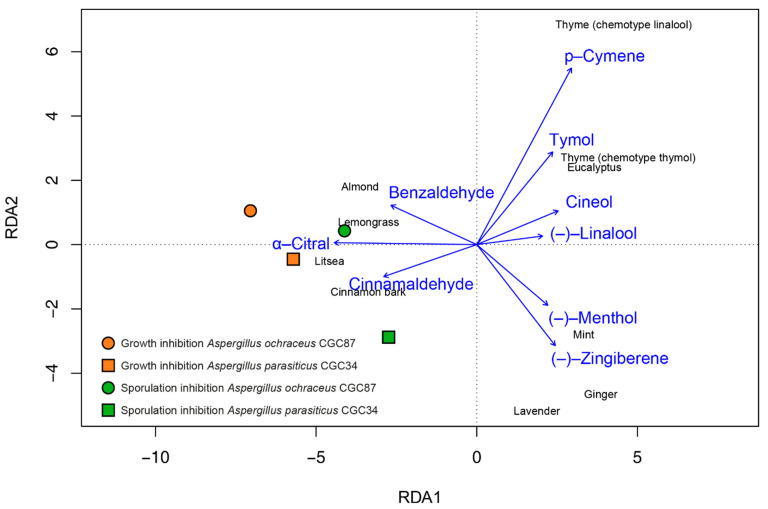
Scatterplot of Redundancy analysis (RDA) showing that the main components of essential oils affect the growth and sporulation of *A. parasitcus* CGC34 and *A. ochraceus* CGC87.

**Figure 2:**
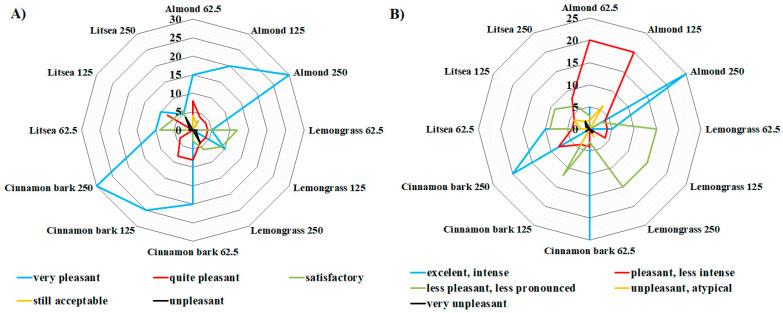
Overall sensory evaluation of the aroma (**A**) and taste (**B**) of coffee samples treated with essential oils (almond, cinnamon bark, lemongrass, and litsea EO) before roasting at a concentration of 62.5, 125, and 250 μL/L (7 evaluators; in the figure, we present only the maximum values that the essential oils obtained during the sensory evaluation).

**Figure 3:**
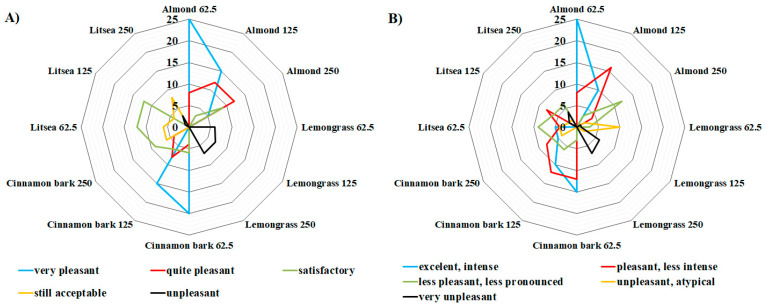
Overall sensory evaluation of the aroma (**A**) and taste (**B**) of coffee samples treated with essential oils (almond, cinnamon bark, lemongrass, and litsea EO) after roasting at concentrations of 62.5, 125, and 250 μL/L (7 evaluators; in the figure, we present only the maximum values that the essential oils obtained during the sensory evaluation).

For each treatment, all the assays were carried out in independent triplicate in this study. The results shown in Table 2 and Table 3 were evaluated by using STASTGRAPHIC Centurion XVI (version 16.1.11)(The Plains, Virginia, USA) (analysis of variance—single-factor and multifactor ANOVA, confidence level 95%, *p <* 0.05) and the homogeneity groups based on the efficiency of tested essential oils were found. Redundancy analysis was performed in the R statistical environment version 4.0.2 [27] using library vegan version 2.5.6 [28] (Figure 1). The obtained point from sensory evaluation for treated coffee samples was displayed in radar graphs (Figure 2 and Figure 3) in points to express differences in the taste and aroma of individual samples.

## 3. Results and Discussion

### 3.1. Essential Oils Effect on the Growth, Sporulation, and Mycotoxins Production of Fungi on Green Coffee Beans

The antifungal (Table 2) and antisporulation activity (Table 3) of essential oils were tested in triplicate for each oil at each concentration. All tested essential oils, except thyme chemotype thymol (0% of growth inhibition), had an inhibitory effect on the growth of *A. parasiticus* CGC34 on the green coffee beans at the lowest concentration (15.625 μL/L) used. When comparing thyme essential oils, regardless of the used concentration, the chemotype thymol EO had a better overall inhibitory effect (48.33%). On the other hand, the chemotype linalool EO at the lowest concentration (15.625 μL/L) had the better inhibitory effect on *A. parasiticus* CGC34 growth (22.22% of growth inhibition). Better results were obtained with the thyme essential oils in previous studies [25], where thyme essential oil was able to inhibit growth of *A. parasiticus* (SLO-B-219) completely at concentrations of 31.25 μL/L (of air) and 62.5 μL/L (of air) [29]. At the highest concentration used (250 μL/L), all essential oils were able to inhibit at least 50% of *A. parasiticus* CGC34 growth. The lowest inhibitory effect on *A. parasiticus* CGC34 growth at this concentration was shown for mint (40% of growth inhibition), eucalyptus (43.33%), and ginger EOs (46.67%). Further, Yooussef et al. [30] tested the antifungal activity of 17 essential oils on the growth of *A. parasiticus* isolated from peanuts at five concentrations (500, 1000, 1500, 2000, 2500 ppm). Cinnamon, lemongrass, and thyme EOs were found to be the most effective essential oils. They had an inhibitory effect at the lowest concentration used (500 ppm) and had a 100% inhibitory effect at concentrations of 1000 ppm (cinnamon), 1500 ppm (lemongrass), and 2500 ppm (thyme), respectively. In their study, mint, ginger, and eucalyptus EOs were less effective, similarly to our study. In contrast, a better inhibitory effect of ginger essential oil was noted by Hussein and Joo [31]; in their study, ginger EO showed a significant inhibitory effect on the growth of all tested species of microscopic filamentous fungi. Ginger EO had a complete (100%) inhibitory effect on the growth of *Cylindrocarpon destructans*, *Sclerotinia sclerotiorum*, and *Sclerotinia nivali* at the concentration of 0.1% *v*/*v*. The highest and completely (0% of infected seeds when treated with these EOs) inhibitory effect on the growth of *A. parasiticus* CGC34 on green coffee beans was recorded for cinnamon bark, lemongrass, almond, and litsea EOs at a concentration 62.5 μL/L (of air). Moreover, the growth of *A. ochraceus* CGC87 was completely inhibited by these EOs (almond, lemongrass, litsea, and cinnamon bark) on coffee beans (0% of coffee beans were infected by fungi) at a concentration of 62.5 μL/L (Table 2). Similar results were obtained by Moghadam et al. [32] with cinnamon EO, where cinnamon showed a higher inhibitory effect on the growth of *A. ochraceus* CBS 263.67 followed by the other essential oils (clove, thyme, cumin, and caraway). Further, Hua et al. [33] found that complete fungal growth inhibition of *A. ochraceus* was obtained at concentrations of 150 to 250 μL/L with contact assays for cinnamon and *Litsea citrate* EOs. However, *A. ochraceus* CGC87 was the most resistant to antifungal activity of tested essential oils. At the lowest tested concentration (15.625 μL/L), ginger, eucalyptus, and thyme essential oils (chemotype thymol) had no inhibitory effect on the growth of this species on green coffee beans. In comparison with *A. parasiticus* CGC34 species, these essential oils were the least effective throughout the cultivation period, regardless of the concentration tested. All essential oils showed the ability to inhibit at least 50% of the growth of *A. ochraceus* CGC87 on infected coffee beans at the highest concentration (250 μL/L) used compared with the control samples. The strong inhibitory activity against the growth of several fungal species, including the genus *Aspergillus,* has been confirmed by many other authors mainly for lemongrass [21,34,35], litsea [36,37,38], and almond [39] essential oils.

Based on the obtained results, we found that all essential oils used in our work had a certain inhibitory effect on *A. parasiticus* CGC34 and *A. ochraceus* CGC87 sporulation even at the lowest concentration tested (Table 3). After statistical evaluation of the obtained results, statistically significant differences between the effect of individual EOs (*p* < 0.05) on the sporulation of *A. parasiticus* CGC34 and *A. ochraceus* CGC87 were found. Cinnamon bark (97.22%), litsea (96.11%), lavender (91.11%), and lemongrass essential oil (90.56%) had the highest overall inhibitory effect on *A. parasiticus* sporulation. Moreover, some of these essential oils, except lavender EO, showed a complete (100%) inhibitory effect at a concentration of 62.5 μL/L (of air). Almond EO was also effective (100% inhibition of spore germination) at this concentration (62.5 μL/L of air), but its overall inhibitory effect was only 88.33% in comparison with other effective EOs. The lower concentrations (15.625 to 31.25 μL/L of air) of the most effective essential oils affected the sporulation of *A. parasiticus* CGC34 ranged from a minimum of 2.78% (coffee beans with the visible sporulation) for litsea essential oil (31.25 μL/L of air) and a maximum of 33.33% (coffee beans with the visible sporulation) for almond essential oil (15.25 μL/L of air). Božik et al. [21] observed the effect of essential oils on the sporulation of *A. parasiticus* (KMi13) on the oat grains. Their results showed that the most effective EOs were lemongrass, oregano, and thyme, at a concentration of 500 μL/L with a complete inhibitory effect on *A. parasiticus* sporulation. In their study, the cinnamon EO inhibited only 73% of *A. parasiticus* (KMi13) spore germination at the highest concentration (250 μL/L). In our study, lemongrass was one of the most effective essential oils; on the contrary, cinnamon bark EO was able to inhibit the sporulation of *A. parasiticus* CGC34 at a concentration lower than 250 μL/L (62.5 μL/L of air) completely (100%). Other essential oils also proved to be effective but, even at the highest concentration tested (250 μL/L of air), they were not 100% effective. Thyme essential oils (chemotype linalool and thymol) were again the least effective, having an inhibitory effect of 55.56% (chemotype linalool) and 66.67% (chemotype thymol). The better results with thyme essential oils on the growth and sporulation of *A. parasiticus* and *A. flavus* isolated from coffee and peanuts after five, seven, and nine days of cultivation were obtained by Silva et al. [40]; they observed the effect of 4 essential oils, namely, ginger, thyme, mint, and fennel. In the case of *A. parasiticus* sporulation, thyme EO showed the better inhibitory effect during all days of cultivation. For the essential oils mint and ginger, a more pronounced inhibitory effect was observed only after the ninth day of cultivation. Moreover, Bluma et al. [41] studied the antisporulation effect of essential oils on the sporulation of *A. parasiticus* (RCT20 and RCD106) and *A. flavus* (RCD65 and RCI105). In *A. parasiticus*, anise EO had the highest inhibitory effect, but mint EO did not show any inhibitory effect. On the contrary, in our case, mint (84.44%) and ginger (87.22%) were two essential oils with a total inhibition of sporulation of *A. parasiticus* CGC34 above 80%. Compared with *A. parasiticus* CGC34, *A. ochraceus* CGC87 was the more sensitive species to the effects of the essential oils with the most potential (almond, lemongrass, cinnamon bark, and litsea), especially to almond EO, which inhibited its sporulation up to 90.00% regardless of the concentration used (Table 2). However, *A. ochraceus* CGC87 again showed the greater resistance to other essential oils. As in the case of *A. parasiticus* CGC34, the least effective essential oils were ginger (overall inhibition of sporulation only 43.89%) and thyme (chemotype linalool) essential oils (overall inhibition of sporulation only 60.56%). Based on the results, we can say that the vapor phase of essential oils effectively inhibits the growth and development of microscopic filamentous fungi on green coffee beans and suppresses their sporulation at the same time. Inouye et al. [42] came to a similar conclusion by testing the antisporulation effect of lemongrass, cinnamon bark, lemon, lavender, thyme, perilla, and tea tree oils. They found that the most potent essential oils in their study was less effective when applied as a solution and antisporulation effects were not observed. However, after exposure of fungal pathogens to essential oils vapors, sporulation of four fungal species—namely, *Aspergillus fumigatus*, *Fusarium solani*, *Penicillium expansum* and *Rhizopus oryzae*—were suppressed. So, the vapors of essential oils were the active forms and can affect growth and sporulation of fungal pathogens. Some of these EOs have also been used in active packaging to preserve the quality of packaged foods, which showed that EOs in the vapor phase provided antifungal capacity [43]

After evaluating the antifungal and antisporulation effect of essential oils, their ability to inhibit production of mycotoxins (AFB_1_, AFG_1_, and OA) were monitored. All coffee beans that were infected with microscopic fungi (only coffee beans with visible fungal growth and sporulation were used for the analysis) were recultivated on fresh SDA medium and cultivated for the next 7 days.

The results show that all 10 essential oils were able to inhibit the production of aflatoxin B_1_ by *A. parasiticus* CGC34 and production of ochratoxin A by *A. ochraceus* CGC87. However, the production of AFG_1_ was not affected by the six essential oils—namely, thyme (both chemotype), eucalyptus, ginger, mint, and lavender—in any case (Table 4). Among the most effective essential oils for inhibiting AFB_1_ and AFG_1_ were lemongrass, cinnamon bark, and litsea EOs. Except cinnamon bark, these essential oils significantly affected the production of both mycotoxins even at the lowest concentration used (15.625 μL/L of air) compared with the control sets. The excellent antitoxigenic potential of these essential oils has been demonstrated in previous studies [20,25,29,44]. Cinnamon bark EO inhibited AFG_1_ production up to a concentration of 31.5 μL/L (of air). Similar results were obtained by El-Aziz et al. [45], who studied the inhibition of growth and production of aflatoxins B_1_, B_2_ and G_1_, G_2_ by *A. parasiticus*, and *A. flavus* in cashew nuts with 5 essential oils (thyme, mint, garlic, cinnamon, and rosemary). Their results showed a significant inhibition in production of AFB_1_ and AFG_1_ with cinnamon EO (67.3—70.7%). In the case of inhibition of G_1_ and G_2_ aflatoxins, this essential oil was also effective, but with a lower-percentage inhibitory effect. Litsea EO was also a very effective essential oil. The production of all mycotoxins (AFB_1_, AFG_1_, and OA) was significantly inhibited by this EO. The antitoxigenic potential of this essential oil was also demonstrated in the study of Li et al. [36] in the inhibition of AFB_1_ production by *A. flavus* (CGMCC 3.4410). Litsea essential oil at a concentration of 1 μL/L (in contact assay) completely (100%) inhibited the production of AFB_1_. In contrary, Foltinová et al. [37] tested litsea EO against the production of cyclopiazonic acid produced by *Penicillium commune* (KMi 177, KMi 270, KMi 276, KMi 277, KMi 370, KMi 402, KMi 403). Litsea had a very low inhibitory effect on cyclopiazonic acid (CPA) production and inhibition occurred only at the highest tested concentration (625 μL/L) of air. Lavender essential oil also showed strong antitoxigenic activity able to inhibit in total (regardless of the concentration used) 76.56% of AFB_1_ production and up to 89.29% of OA production (calculated from the number of infected/production strains). In this study, almond oil was the only effective essential oil that did not inhibit aflatoxin production at concentration 15.625 μL/L (of air). However, production of OA by *A. ochraceus* CGC87 was affected by almond essential oil at this concentration up to over 50% (among the 12 tested coffee beans (with visible fungal growth and sporulation), the production of OA was detected only in 5 cases). The least effective was ginger EO, which inhibited only 9.36% of AFB_1_ production, regardless of concentration used (among the 96 tested coffee samples, the production of AFB_1_ was detected in up to 87 cases). In the case of OA inhibition, its effect was better but only at the highest concentration used. Overall (regardless of concentration used), ginger EO inhibited only 13.56% of OA production. The effect of 5 essential oils (cinnamon, turmeric, basil, ginger, and palmarosa essential oils) on the inhibition of growth and ochratoxin A production by *Aspergillus ochraceus* (ITCC 1456) and *Penicillium verrucosum* (ITCC 2986) on maize grains was observed by Kalagatur et al. [46]. In their study, all essential oils showed an inhibitory effect. The best effect (complete inhibition) on the production of OA was obtained in treatment with cinnamon and palmarosa EOs in both species. In both cases, ginger EO was the least effective, which in our case, was one of the less-effective essential oils for inhibiting aflatoxins. Noshirvani and coauthors [47] obtained similar results with chitosan films containing cinnamon and ginger EOs. In their study, the films containing cinnamon oil showed higher antifungal activity against *Aspergillus niger* than those containing ginger in in vitro condition. The results showed that the effect of essential oils on fungal growth, sporulation, and mycotoxins production is influenced not only by the type of essential oil tested, but also by the origin of the fungal strain used in the study.

The presence of mycotoxins in coffee beans can have adverse effects on consumer health. Garcia-Moraleja et al. [48] investigated the presence of 21 mycotoxins, including AFs and OA, in coffee. The results showed that all studied mycotoxins were found in the samples at concentrations from 0.69 μg/kg to 282.89 μg/kg. However, according to their study, coffee intake does not pose a potential risk to coffee consumers. On the contrary, Cramer et al. [49] studied human exposure to the mycotoxin ochratoxin A (OA) and its thermal degradation product 2′R-ochratoxin A through blood samples from coffee and noncoffee drinkers. Comparison between coffee and noncoffee consumers showed that 2′R-OA (at concentrations from 0.11 g/L to 0.414 g/L) was only present in blood from the first group while OA could be found in both groups (0.21 g/L). This finding could be related to the consumption of other OA-contaminated foods. Due to the different results of the mentioned studies above, the presence and levels of mycotoxins in coffee need to be controlled to avoid potential risks to consumers’ health.

### 3.2. Chemical Analysis of Essential Oils

Among the most effective essential oils capable of inhibiting the growth, sporulation, and production of mycotoxins on green coffee beans were almond, lemongrass, cinnamon bark, and litsea. Using Redundancy analysis, we identified the main components describing variation in composition of essential oils and the main components that affected growth and sporulation of *A. parasiticus* CGC34 and *A. ochraceus* CGC87 (Figure 1). According to the analysis, Benzaldehyde a Citral and Cinnamaldehyde were associated with the highest inhibition of growth and sporulation.

The content of the major chemical components of the essential oils and authentic standards used in this study are summarized in Table 5. The main and, at the same time, the only component represented in almond essential oil was benzaldehyde (98.20%). The excellent antifungal effects of benzaldehyde and some of its derivatives have been confirmed in several studies [39,50,51,52]. Its antifungal potential probably lies in its ability to disrupt cellular antioxidant systems and, thus, inhibit fungal growth [51]. Cinnamon bark was another very effective essential oil with the highest proportion of cinnamaldehyde (65.30%). This compound is very effective in inhibiting the growth of fungal pathogens, especially of the genus *Aspergillus*. Treatment of *A. flavus* with Cinnamaldehyde showed a decrease in spore germination [53] and retarding of the hyphal elongation of *A. niger* [54], causing alterations in cellular morphology and damage to cell wall and plasma membrane [55,56] or can inhibit the production of aflatoxins [57]. The last essential oils with high antifungal or antitoxigenic activity were litsea and lemongrass with a high content of α–Citral (39.00% for litsea EO and 37.15% for lemongrass EO) and β–Citral (33.37% for litsea EO and 39.27% for lemongrass EO). The vapors of Citral, its isomers Geranial and Neral, can disrupt fungal mycelium [58], reduce membrane permeability [59], damage mitochondria, and induce cell death [60], inhibiting ergosterol biosynthesis or mycotoxin production, such as ochratoxin A [33]. Lavender EO was not one of the most effective essential oils, but it showed a significant ability to inhibit AFB_1_ and OA production and to suppress the sporulation of *A. parasiticus* CGC34 (91.11%). It is characterized by a particularly high content of (–)–Linalool (40.52%), which is described as active especially against yeasts, where it penetrates rapidly into yeast cells, causing extensive lesions of the plasma membrane and a significant reduction in ergosterol [61]. Thyme EO (chemotype linalool) was also characterized by a higher content of (–)–Linalool (32.00%), but its antifungal effects were not as pronounced as with lavender essential oil. This fact could be related to the minor components present in the essential oils, which can act either synergistically or antagonistically.

### 3.3. Sensory Evaluation of Roasted Coffee

Essential oils have real potential for the food industry in various areas. Therefore, they can be used as preservatives in various foods, such as meat and meat products, vegetables and fruits, dairy products, and others [62]. However, despite their wide biological properties (antibacterial, antifungal, antioxidant, etc.), their use in the food industry is limited. One of the main obstacles facing the food industry in relation to the use of essential oils and their bioactive components is their intense aroma and subsequent interactions with food matrix. As a result, it is possible to apply only certain doses of essential oils to foods, which at the same time still have the desired inhibitory effect on the growth and multiplication of pathogens causing food contamination. However, to achieve this effect, a much higher concentration of essential oils is usually needed, and this can have a negative impact on the organoleptic and sensory properties of food [63]. In general, the most important aspects of sensory analysis of essential-oil-treated foods are their visual quality, color, taste, and smell [64].

For this reason, in this work, the impact of EO vapors on coffee beans were evaluated. The seven untrained evaluators assessed the sensory properties of coffee beverage prepared from coffee beans treated with 4 selected essential oils, namely, almond, cinnamon bark, lemongrass, and litsea. Three concentrations of EOs (62.5, 125, and 250 μL/L of air) were used. For sensory analysis, the EOs and their concentrations were selected based on their inhibitory effect on growth, sporulation, and mycotoxin production of both tested fungal species (*A. parasiticus* CGC34 and *A. ochraceus* CGC87). Each tested EO could obtain a maximum of 35 points and a minimum of 7 points in evaluating their impact on the organoleptic properties (taste and smell) of coffee beverage. The results showed that none of the tested essential oils scored the maximum number of points (35 points) in any of the indicators in the evaluation of taste and odor, respectively. For this reason, and for the sake of clarity, the maximum possible value (35 points) that the essential oils could obtain in a positive evaluation is not shown in Figure 2 and Figure 3.

First, coffee beans that were roasted before treatment with essential oils were evaluated. All panel members were able to determine the specific tastes and odors for the tested essential oils in coffee beverages. Due to the similar chemical composition of lemongrass and litsea EOs, the samples treated with these oils obtained similar scores in taste and odor evaluation, respectively. Odor testing (Figure 2A) showed that samples treated by almond and cinnamon bark EOs proved to be the most acceptable for consumers at all tested concentrations (62.5, 125, and 250 μL/L of air). At the highest tested concentration (250 μL/L air), both essential oils had a high evaluation score (30 points for both EOs) for a very pleasant effect on the aroma of the coffee beverage. Lemongrass and litsea EOs appeared to be the least acceptable at this concentration, and coffee treated with these essential oils received the most points (4 points for both EO) in unpleasantness ranking. Similar results with lemongrass essential oil were obtained in a previous study [25]. The effect of thyme, clove, cinnamon, oregano, cumin, and lemongrass essential oils on the sensory properties of treated bread was analyzed. Differences in sensory properties were observed for oregano and lemongrass essential oil, especially at higher concentrations (250 and 500 μL/L of air). Oregano caused a pungent taste of bread and lemongrass had an unpleasant lemon taste, so they were evaluated as unacceptable. Other essential oils did not have a significant effect on the organoleptic properties of the bread. In contrast, in a study by Božik et al. [21], a sensory evaluation of the essential oils effect on the organoleptic properties of oats, lemongrass EO was rated as the best and most suitable for treatment by essential oil. Different results suggest that not every meal tolerates every type of essential oil well.

All samples treated with essential oils were also evaluated for intensity and acceptability of their taste. Essential oils with a citrus scent were again rated as the worst because they caused the sour taste of coffee drinks. Based on the results of the evaluated essential oils, almond EO with 25 points (250 μL/L air) proved to be the most acceptable, which gave the coffee a pleasant, less-intense taste (Figure 2B). All other essential oils were generally rated as less pleasant and less pronounced.

The sensory evaluation of the taste and aroma of coffee beverages samples prepared from green coffee beans treated with essential oils before roasting coffee received a low rating. These results could be related to the fact that the green coffee beans changed their moisture content after treatment with EOs before roasting. Therefore, it could indicate that the essential oils chemically bound to the coffee beans and, thus, caused a more intense taste and aroma of the resulting coffee beverages. All coffee samples treated with essential oils at concentrations above 62.5 μL/L (air) were evaluated as satisfactory, still acceptable, or unpleasant in the odor evaluation. Only in the case of coffee samples treated with almond and cinnamon bark EOs were they evaluated as very pleasant or quite pleasant at concentrations from 62.5 to 125 μL/L (air) (Figure 3A). The worst rating in the aroma was given to the lemongrass EO, whose penetrating citrus aroma was atypical in coffee. In the evaluation of taste, the coffee samples treated with this EO were marked as the worst. Samples of coffee beverages treated with almond essential oil at a concentration of 62.5 μL/L (of air) were evaluated as the best with a score of 25 points in the evaluation of excellent intense taste (Figure 3B). Similar results were obtained by Laranjo et al. [65]. They evaluated acceptability of the taste and aroma of some food samples (goat cheese, meat (pork sausages), strawberries, and grapefruit) treated with various concentrations of essential oils (cinnamon, thyme, clove, rosemary, oregano, and sage). In sensory analysis of the fruits, the application of essential oils proved to be acceptable, but the use of a higher concentration for the already treated fruit appeared to be unacceptable. Oregano EO was excluded from the goat cheese analysis because it caused an unsuitable bitter taste. In sensory analysis of meat, especially sausages, the essential oils tested were also acceptable.

For this reason, it is very important to choose the right combination of essential oils and the foods that will be treated with them. Some spices are still used in the preparation of various dishes and improve their taste, such as oregano or rosemary in the preparation of lamb or fish meat. In our case, cinnamon bark and almond EOs did not have a significant negative effect on the taste or aroma of coffee drinks; on the contrary, in certain concentrations, they improved their taste and aroma.

## 4. Conclusions

In conclusion, we found that the vapor phase of four essential oils—namely, litsea, almonds, cinnamon bark, and lemongrass—had a complete (100%) inhibitory effect on the growth of both tested fungal species (*A. parasiticus* CGC34 and *A. ochraceus* CGC87) with origin of green coffee beans at a concentration of 62.5 μL/L of air. Cinnamon bark essential oil showed the highest sporulation inhibitory effect for both species (97.22% for *A. parasiticus* CGC34 and 97.78% for *A. ochraceus* CGC87). Litsea EO showed the strongest antitoxigenic activity against aflatoxins and ochratoxin A production. In addition, sensory analysis showed that cinnamon bark and almond EO do not have a significant negative effect on the organoleptic properties (taste and odor) of coffee samples that have been treated with them and accepted by consumers. Thus, our findings suggest that these essential oils (cinnamon bark and almond) are highly effective in the vapor phase and could be used to protect coffee beans from degradation by toxigenic fungi. However, for proper use, it is still important to find the right combination of food and essential oil.

## Figures and Tables

**Table 1:** Genbank accession numbers for ITS (Internal Transcribed Spacer) genes for the used *Aspergillus* strain and their ability to produce mycotoxins.

Fungal Strains Used in Study	Genbank Accession Numbers (ITS)	Mycotoxins Produced by Strains *
*Aspergillus parasitcus* CGC34	OL449522	Aflatoxin B_1_, Aflatoxin G_1_
*Aspergillus ochraceus* CGC87	OL449521	Ochratoxin A

Legend: * the ability to produce mycotoxins was confirmed by thin layer chromatography (TLC).

**Table 2:** Percentage of infected green coffee beans by toxigenic aspergilli in EOs treatments with concentration range from 15.625 to 250 (μL/L of air) (for all tested EOs and concentrations, *n* = 36) in multiple range test (95.0% Tukey HSD (Honestly Significant Difference)) after 7 days of cultivation.

Essential Oils and Control Sets	Tested Concentrations of EOs (μL/L of Air)					Inhibition of Fungal Growth (%) on Green Coffee Beans (Average)
	250	125	62.5	31.25	15.625	
	*Aspergillus parasiticus* (CGC34) *					
Almond	0 ^a^	0 ^a^	0 ^a^	25.00 ^a^	41.67 ^a^	86.67 ^a^
Lemongrass	0 ^a^	0 ^a^	0 ^a^	27.78 ^a^	33.33 ^a^	87.78 ^a^
Thyme (chemotype linalool)	30.56 ^bc^	33.33 ^b^	50.00 ^bc^	69.44 ^bc^	77.78 ^bc^	47.78 ^c^
Thyme (chemotype thymol)	11.11 ^ab^	30.56 ^b^	50.00 ^bc^	66.67 ^b^	100 ^c^	48.33 ^c^
Eucalyptus	41.67 ^c^	41.67 ^bc^	58.33 ^bc^	66.67 ^b^	75.00 ^bc^	43.33 ^c^
Ginger	16.67 ^ab^	41.67 ^bc^	50.00 ^bc^	75.00 ^bc^	83.33 ^c^	46.67 ^c^
Mint	33.33 ^bc^	50.00 ^b^	66.67 ^c^	75.00 ^bc^	75.00 ^bc^	40.00 ^c^
Cinnamon bark	0 ^a^	0 ^a^	0 ^a^	25.00 ^a^	33.33 ^a^	88.33 ^a^
Lavender	27.78 ^bc^	33.33 ^b^	33.33 ^b^	30.56 ^a^	50.00 ^ab^	65.00 ^b^
Litsea	0 ^a^	0 ^a^	0 ^a^	13.89 ^a^	41.67 ^a^	88.89 ^a^
Growth control	100 ^d^	100 ^c^	100 ^d^	100 ^c^	100 ^c^	0 ^d^
	*Aspergillus ochraceus* (CGC87) *					
Almond	0 ^a^	0 ^a^	0 ^a^	25.00 ^a^	33.33 ^a^	88.33 ^a^
Lemongrass	0 ^a^	0 ^a^	0 ^a^	25.00 ^a^	36.11 ^a^	87.78 ^a^
Thyme (chemotype linalool)	33.33 ^bc^	33.33 ^b^	50.00 ^b^	66.67 ^b^	75.00 ^bc^	48.33 ^b^
Thyme (chemotype thymol)	13.89 ^ab^	41.67 ^bc^	58.33 ^b^	100 ^c^	100 ^c^	37.22 ^bc^
Eucalyptus	22.22 ^abc^	44.44 ^bc^	75.00 ^bc^	91.67 ^bc^	100 ^c^	33.33 ^c^
Ginger	19.44 ^abc^	41.67 ^bc^	75.00 ^bc^	91.67 ^bc^	100 ^c^	34.44 ^c^
Mint	41.67 ^c^	66.67 ^c^	75.00 ^bc^	91.67 ^bc^	91.66 ^c^	26.67 ^c^
Cinnamon bark	0 ^a^	0 ^a^	0 ^a^	30.56 ^a^	50.00 ^ab^	83.89 ^a^
Lavender	27.78 ^bc^	50.00 ^bc^	50.00 ^b^	91.67 ^bc^	91.66 ^c^	37.78 ^bc^
Litsea	0 ^a^	0 ^a^	0 ^a^	16.67 ^a^	33.33 ^a^	90.00 ^a^
Growth control	100 ^d^	100 ^d^	100 ^c^	100 ^c^	100 ^c^	0 ^d^

Legend: * strain ID; data in the column followed by same letters represent homogeneity between different groups of essential oils and no statistically significant differences (*p* > 0.05); different letters represent statistically significant differences in 95.0% Tukey HSD test (*p* > 0.05); Growth control—green coffee beans without essential oil treatment.

**Table 3:** Percentage of fungal sporulation on green coffee beans treated by EOs with concentration range from 15.625 to 250 (μL/L of air) (for all tested EOs and concentrations, *n* = 36) in multiple range test (95.0% Tukey HSD) after 7 days of cultivation.

Essential Oils and Control Sets	Tested Concentrations of EOs (μL/L of Air)					Inhibition of Fungal Sporulation (%) on Green Coffee Beans (Average)
	250	125	62.5	31.25	15.625	
	*Aspergillus parasiticus* (CGC34) *					
Almond	0 ^a^	0 ^a^	0 ^a^	25.00 ^abc^	33.33 ^bc^	88.33 ^ab^
Lemongrass	0 ^a^	0 ^a^	0 ^a^	22.22 ^abc^	25.00 ^ab^	90.56 ^ab^
Thyme (chemotype linalool)	22.22 ^c^	33.33 ^c^	41.67 ^d^	55.56 ^d^	69.44 ^d^	55.56 ^d^
Thyme (chemotype thymol)	5.56 ^a^	11.11 ^ab^	30.56 ^bcd^	50.00 ^cd^	69.44 ^d^	66.67 ^cd^
Eucalyptus	8.33 ^ab^	8.33 ^ab^	33.33 ^cd^	44.44 ^bcd^	47.22 ^c^	67.78 ^c^
Ginger	2.78 ^a^	22.22 ^bc^	16.67 ^abc^	16.67 ^ab^	19.44 ^ab^	87.22 ^ab^
Mint	8.33 ^ab^	11.11 ^ab^	16.67 ^abc^	19.44 ^ab^	22.22 ^ab^	84.44 ^b^
Cinnamon bark	0 ^a^	0 ^a^	0 ^a^	5.56 ^a^	8.33 ^a^	97.22 ^a^
Lavender	5.56 ^a^	8.33 ^ab^	8.33 ^ab^	11.11 ^a^	11.11 ^a^	91.11 ^ab^
Litsea	0 ^a^	0 ^a^	0 ^a^	2.78 ^a^	16.67 ^ab^	96.11 ^a^
Growth control	100 ^d^	100 ^d^	100 ^e^	100 ^e^	100 ^e^	0 ^e^
	*Aspergillus ochraceus* (CGC87) *					
Almond	0 ^a^	0 ^a^	0 ^a^	19.44 ^abc^	30.56 ^ab^	90.00 ^b^
Lemongrass	0 ^a^	0 ^a^	0 ^a^	16.67 ^abc^	27.78 ^ab^	91.11 ^ab^
Thyme (chemotype linalool)	16.67 ^ab^	30.56 ^b^	41.67 ^b^	41.67 ^c^	66.67 ^cd^	60.56 ^e^
Thyme (chemotype thymol)	8.33 ^ab^	19.44 ^ab^	41.67 ^b^	41.67 ^c^	47.22 ^bc^	68.33 ^d^
Eucalyptus	5.56 ^ab^	11.11 ^ab^	25.00 ^ab^	33.33 ^bc^	41.67 ^bc^	76.67 ^c^
Ginger	19.44 ^b^	27.78 ^b^	44.44 ^b^	91.67 ^d^	97.22 ^de^	43.89 ^f^
Mint	13.89 ^ab^	19.44 ^ab^	19.44 ^ab^	27.78 ^abc^	30.56 ^ab^	77.78 ^c^
Cinnamon bark	0 ^a^	0 ^a^	0 ^a^	2.78 ^a^	8.33 ^a^	97.78 ^a^
Lavender	13.89 ^ab^	25.00 ^b^	25.00 ^ab^	30.56 ^abc^	69.44 ^cde^	67.22 ^de^
Litsea	0 ^a^	0 ^a^	0 ^a^	5.56 ^ab^	8.33 ^a^	97.22 ^ab^
Growth control	100 ^c^	100 ^c^	100 ^c^	100 ^d^	100 ^e^	0 ^g^

Legend: * strain ID; data in the column followed by same letters represent homogeneity between different groups of essential oils and no statistically significant differences (*p* > 0.05); different letters represent statistically significant differences in 95.0% Tukey HSD test (*p* > 0.05); Growth control—green coffee beans without essential oil treatment.

**Table 4:** In situ inhibitory effects of essential oils at 22 ± 1 °C after 7 days of cultivation on the mycotoxin production by *Aspergillus* spp. (three replications in treatments with each essential oil at each tested concentration were screened (12 inoculated coffee beans in three repetitions (*n* = 36)).

Essential Oils and Control Sets	Tested Concentrations of EOs (μL/L of Air)									
	250		125		62.5		31.25		15.625	
	*Aspergillus parasiticus* (CGC34) *									
	AFB_1_	AFG_1_	AFB_1_	AFG_1_	AFB_1_	AFG_1_	AFB_1_	AFG_1_	AFB_1_	AFG_1_
Almond	NA	NA	NA	NA	NA	NA	9/6	9/3	15/15	15/15
Lemongrass	NA	NA	NA	NA	NA	NA	10/1	10/4	12/2	12/5
Thyme (l)	11/5	11/11	12/9	12/12	18/11	18/12	25/20	25/25	28/25	28/28
Thyme (t)	4/2	4/4	11/5	11/11	18/9	18/18	25/20	25/25	36/30	36/36
Eucalyptus	15/5	15/15	15/7	15/15	21/11	21/21	24/14	24/24	27/17	27/27
Ginger	6/2	6/6	15/13	15/15	18/15	18/18	27/27	27/27	30/30	30/30
Mint	12/2	12/12	18/8	18/18	24/14	24/24	27/17	27/27	27/17	27/27
CB	NA	NA	NA	NA	NA	NA	9/1	9/6	12/2	12/12
Lavender	10/1	10/10	12/2	12/12	12/2	12/12	12/2	12/12	18/8	18/18
Litsea	NA	NA	NA	NA	NA	NA	5/1	5/1	15/5	15/5
GC (NT)	36/36									
	*Aspergillus ochraceus* (CGC87) *									
	OA									
Almond	NA		NA		NA		9/3		12/5	
Lemongrass	NA		NA		NA		9/1		13/4	
Thyme (l)	12/8		12/6		18/85		24/8		27/14	
Thyme (t)	5/2		15/3		21/6		36/7		36/8	
Eucalyptus	8/2		16/3		27/4		33/6		36/8	
Ginger	7/3		15/3		27/27		33/33		36/36	
Mint	15/2		24/4		27/4		33/5		33/6	
CB	NA		NA		NA		12/1		18/1	
Lavender	10/1		18/2		18/2		33/3		33/4	
Litsea	NA		NA		NA		6/1		12/1	
GC (NT)	36/36									

Legend: *—ID of tested strain, conc.—concentration, NA—not analyzed (no visible growth of colony on coffee beans), X/Y—number of tested coffee beans infected by fungal strains/numbers of positive fungal strains, OA—ochratoxin A, AFB_1_/AFG_1_—aflatoxins, GC—growth control; NT—not treated with essential oils; Thyme (l)—chemotype linalool; Thyme (t)—chemotype tymol, CB—cinnamon bark.

**Table 5:** Major components of essential oils determined by GC–MS and quantified by GC–FID techniques and authentic standards used in this study.

RI ^b^		Component	Tl ^c^*	Tt	M	E	CB	LC	G	LG	A	L
940	^a^	(+)–α–pinene	2.50	1.00	0.66	2.88	0.16	1.46	1.11	0.52		1.13
954	^a^	Camphene	0.84	1.15					3.49	0.70		0.69
964	^a^	Benzaldehyde									98.20	
980	^a^	(–)–β–Pinene	0.50	0.43	0.81	0.30		1.12		1.27		0.83
982		1-Octen-3-ol										0.42
989		Methylheptenone						0.88				
993		β–Myrcene	0.80	1.53					0.29			0.89
998		butylisothiocyanate				0.71						
1006	^a^	α–Phellandrene				0.33	0.14		0.17			
1020	^a^	α–Terpinene		0.70		0.10						0.15
1029	^a^	p–Cymene	39.10	18.36		6.30	0.54					0.58
1031		β–Phellandrene						1.61				
1033	^a^	(R)–(+)–Limonene	0.80		1.80	6.00	0.49	14.50	3.76	11.50		1.34
1035	^a^	Cineol	0.72	1.12	8.16	80.01		1.50	1.56			12.01
1043		β–trans–Ocimene				0.10						
1063	^a^	γ–Terpinene		6.44		2.45				0.89		0.14
1076		Linalool oxide										0.51
1101	^a^	(–)–Linalool	32.00	5.10			1.90	1.00	0.30	0.83		40.59
1147	^a^	(−)–Isopulegol		1.73								4.62
1158	^a^	(+/–)–citronellal			22.09			0.67				
1169	^a^	(–)–Borneol	0.82	1.55	8.40			0.63	0.67			7.43
1171	^a^	Lavandulol										3.36
1177	^a^	(–)–Menthol			41.84							
1180		Menth–1–en–4–ol		1.63	1.70							
1192	^a^	4–Terpineol	1.10						0.30			6.93
1198		α–Terpineol										2.40
1238		O–Methyl thymol		0.47								
1245	^a^	β–Citral			0.50			33.37		29.27		
1247	^a^	(–)–carvone		0.82								
1259	^a^	Geraniol						0.89		4.97		
1260		Linalyl anthranilate										10.15
1272	^a^	Cinnamaldehyde					65.30					
1275		α–Citral						39.00		37.15		
1287	^a^	Bornyl acetate										0.12
1293	^a^	2–Undecanone			5.72							
1298	^a^	Tymol	12.11	53.40								
1307	^a^	Carvacrol	0.72	2.56								
1361	^a^	Eugenol	0.89				21.03					
1376		Copaene							0.50			
1386	^a^	Geranyl acetate								4.20		0.26
1419	^a^	β–Caryophyllene	0.90		3.44		4.16	0.99		2.32		
1452	^a^	α–Caryophyllene					0.73					0.50
1478		Germacrene D							0.73			
1481		γ–Muurolene							1.32			
1485		α–Curcumene							14.20			
1490		β–Selinene							1.68			
1497		(–)–Zingiberene							44.36			
1510	^a^	α–Farnesene							12.40	2.00		
1515		γ–Cadinene										0.35
1526		Sesquiphellandrene							11.41			
1574		Caryophyllene oxide										0.46
		total	98.90	97.99	95.12	99.18	94.45	97.62	98.25	95.62	98.20	95.86

Legend: ^a^—Identification confirmed with authentic standard; ^b^—RI, identification based on Kovat’s retention indices (HP–5MS capillary column) and mass spectra; ^c^—Relative proportions were calculated in % by dividing individual peak area by total area of all peaks; * T(linalool)—thyme chemotype linalool; T(thyme)—thyme chemotype thyme; M—mint; E—eucalyptus; CB—cinnamon bark; Li—litsea; G—ginger; LG—lemongrass; A—almond; L—lavender.

## Data Availability

All data are available upon request on corresponding author.
